# Telemedizinische Narkoseaufklärung – Sind Patienten bereit dafür? Eine vergleichende Bedarfsanalyse vor und während der Pandemie

**DOI:** 10.1007/s00101-024-01387-4

**Published:** 2024-02-16

**Authors:** A. Follmann, J. Wienhold, A. Arnolds, M. Derwall, R. Rossaint, M. Czaplik

**Affiliations:** 1 Uniklinik RWTH Aachen, Klinik für Anästhesiologie, Pauwelsstr. 30, 52074 Aachen, Deutschland; 2 Klinik für Anästhesiologie und Operative Intensivmedizin, St. Johannes Hospital Dortmund, Dortmund, Deutschland; 3 Docs in Clouds TeleCare GmbH, Aachen, Deutschland

**Keywords:** Narkose, Anamnese, Digitalisierung, Telemedizin, Pandemie, Anesthesia, Anamnesis, Digitalization, Telemedicine, Pandemic

## Abstract

**Hintergrund:**

Die umfassende Narkosevorbereitung mittels Anamnese und körperlicher Untersuchung gilt als essenzieller Bestandteil der Qualitätskriterien für eine Narkose. Allerdings ist eine Narkosevorbereitung in der Klinik häufig mit langen Wartezeiten in der Anästhesie-Ambulanz verbunden. Zudem sind regelhaft Wiedervorstellungen aufgrund von fehlenden oder noch ausstehenden Befunden erforderlich. Besonders im Rahmen der COVID-19-Pandemie schien die Implementierung von Telemedizin im präoperativen Setting der Anästhesiologie vielversprechend und sinnvoll.

**Ziel der Arbeit:**

Diese vergleichende Querschnittstudie soll über eine Patientenbefragung aufzeigen, für welche Patientengruppen eine telemedizinische Narkosevorbereitung geeignet ist, und welche technischen Rahmenbedingungen auf Patientenseite vorhanden sind.

**Material und Methoden:**

Hierzu wurden anhand eines Fragebogens insgesamt 2080 Patienten (1030 vor, 1050 während der Pandemie) befragt. Für „matched-pairs“-Analysen (Paaranalysen) wurden 630 Paare entsprechend ihres Alters und Geschlechts gebildet.

**Ergebnisse:**

Während der Pandemie nutzten deutlich mehr Patienten die Möglichkeit der Videotelefonie in ihrem Alltag (30,4 % vs. 41,8 %). Vor der Pandemie bewerteten 31,7 % der Patienten die Videotelefonie als praktische und geeignete Methode für ein Aufklärungsgespräch. Nach der Pandemie stieg diese Zahl der Patienten, die gegenüber einer Videotelefonie aufgeschlossenen sind, auf 46,6 % an. Für die Mehrheit der Patienten war der persönliche Kontakt zu einem Anästhesisten vor Ort wichtig (80,7 % vor vs. 67,4 % nach der Pandemie). Die Zahl der Patienten, die über die notwendige technische Ausstattung für eine Videokommunikation verfügten, stieg ebenfalls infolge der COVID-19-Pandemie (50,4 % vs. 58,2 %).

**Diskussion:**

Fast die Hälfte der Patienten scheint bereits heute einer telemedizinischen Narkoseaufklärung offen gegenüberzustehen. Es ist davon auszugehen, dass in Zukunft auch ältere Menschen zunehmend die für eine telemedizinische Narkosevorbereitung erforderliche Technik sowie die erforderliche technische Kompetenz besitzen werden. Bei der Implementierung einer telemedizinischen Narkosevorbereitung sollte die Nutzerakzeptanz zentrales Ziel der Konzeptentwicklung sein. Randomisierte kontrollierte Studien können die Potenziale belegen und mögliche Probleme aufzeigen.

## Abstract (en)

**Background:**

Comprehensive anesthesia preparation by means of the anamnesis and physical examination is considered an essential part of the quality criteria for anesthesia. Especially due to the shortage of specialists, there are usually long waiting times in anesthesia outpatient departments and patients must frequently return in cases of missing or pending findings. Telemedicine already offers alternatives in the context of video communication. These alternatives are now particularly prominent due to the currently existing COVID-19 pandemic and the resulting recommendations for digitalization.

**Objectives:**

This comparative cross-sectional study was carried out to show via a patient survey which patient groups are suitable for a telemedical anesthesia preparation and whether the patients are already technically sufficiently equipped.

**Material and methods:**

For this purpose, a total of 2080 patients (1030 before and 1050 during the pandemic) were interviewed using a questionnaire. For matched paired analyses, 630 pairs were formed according to their age and gender.

**Results:**

Before and after the pandemic, there was an increase in the percentage of patients already using video communication in their daily lives (30.4% vs. 41.8%). Before the pandemic, 31.7% of patients indicated that they considered this concept of communication to be a practical and appropriate method for an educational conversation and after the pandemic this number increased to 46.6%. For the majority of patients personal contact with a local anesthesiologist was important (80.7% before vs. 67.4% during the pandemic). The number of patients who had the necessary technical equipment for video communication also increased as a result of the COVID-19 pandemic (50.4% vs. 58.2%).

**Discussion:**

Almost half of the patients already seem to be open to a telemedical preoperative evaluation. As digitalization progresses, older generations are more likely to recognize the benefits and be able to own and use the necessary technology in the near future. User acceptance should be the central goal of concept development. This must be followed by a randomized controlled study to evaluate the potentials but also the problems in the perioperative process.

## Einleitung

Eine umfassende Narkosevorbereitung beinhaltet neben einer Anamneseerhebung, einer körperlichen Untersuchung und einer Risikobewertung auch die individuelle Aufklärung des Patienten über den Narkoseverlauf, alternative Verfahren und mögliche Komplikationen [1]. Die Narkosevorbereitung ist daher essenzieller Bestandteil der Qualitätskriterien für eine Narkose und gilt als wichtige vertrauensbildende Maßnahme [2]. Sie ist unter medikolegalen Aspekten unverzichtbar für jeden relevanten medizinischen Eingriff.

Auch bedingt durch den Fachkräftemangel kommt es in anästhesiologischen Ambulanzen häufig zu langen Wartezeiten. Berufstätige Patienten müssen zudem Anreise und Gespräch mit ihrem Berufsalltag koordinieren oder gar freinehmen. Dabei können gerade junge und gesunde Patienten zügig befragt und über ein Narkoseverfahren aufgeklärt werden, da nur wenige Vorerkrankungen berücksichtigt und kaum Risiken beschrieben werden müssen [3]. Diesem effektiv kurzen Zeitraum der eigentlichen Aufklärung stehen lange Anfahrt- und Wartezeiten gegenüber.

Bei älteren Patienten ist es für den aufklärenden Anästhesisten von besonderer Bedeutung, dass alle Vorbefunde vorliegen. Häufig fehlen jedoch relevante Informationen, und es müssen zusätzliche Konsile sowie vorhandene Befunde nachgefordert werden. Dies ist oftmals mit einer Wiedervorstellung in der anästhesiologischen Ambulanz verbunden, gerade für ältere Menschen ein beschwerlicher Weg. Würde vor dem Termin ein Abgleich der erforderlichen mit den vorhandenen Befunden erfolgen, könnte man dieser Patientengruppe Anreisen und Wiedervorstellungen ersparen. Für Patienten ohne relevante Vorerkrankungen kann die telemedizinische Narkosevorbereitung somit eine Vorstellung in der Klinik ersetzen, während sie für multimorbide Patienten als Vorbereitung der eigentlichen Vorstellung dienen kann.

Die technischen Voraussetzungen zur Umsetzung einer telemedizinischen Narkosevorbereitung sind bereits gegeben. So kann die Anamnese mittels digitaler Fragebogen erhoben und elektronisch an den Anästhesisten übertragen werden [4]. Die Aufklärung kann mittels moderner Medien im Rahmen der Videokommunikation erfolgen und beispielsweise durch erklärende Videos ergänzt werden [5]. Tatsächlich zeigte eine in Deutschland durchgeführte Studie, dass die videogestützte Patientenaufklärung zu einer signifikant verbesserten Information des Patienten führen kann. Laut den an der Umfrage teilnehmenden Anästhesisten waren die Hauptvorteile eines solchen Ansatzes (a) eine bessere Information der Patienten, (b) die Reduzierung des Zeitaufwands für die präoperative Aufklärung und die körperliche Untersuchung und schließlich (c) die Reduzierung der Arbeitsbelastung der Ärzte [5]. Das individuelle Aufklärungsgespräch kann telemedizinisch mittels Videotelefonie erfolgen. Offene Fragen können so auch über weite Distanzen beantwortet werden.

Bereits 2019 hat die Deutsche Gesellschaft für Anästhesiologie und Intensivmedizin e.V. (DGAI) die Förderung der Telemedizin auch im perioperativen Kontext als Zukunftsstrategie empfohlen [6]. Im Rahmen des Joint Statement des American College of Surgeons mit der American Society of Anesthesiologists sowie weiterer Fachgesellschaften wurde zuletzt die Empfehlung ausgesprochen, Telemedizin auch zur Vorbereitung elektiver Eingriffe nach der COVID-19-Pandemie zu nutzen [7]. Weltweit kommt die Telemedizin aktuell v. a. dann präoperativ zum Einsatz, wenn Patienten bereits im Krankenhaus stationär sind und dort z. B. von einer Pflegekraft untersucht werden können [8]. Kürzlich wurde eine Studie aus Los Angeles veröffentlicht, die telemedizinische Narkosevorbereitungen evaluierte und von Zeit- und Kosteneinsparungen sowie einer hohen Zufriedenheit der Patienten berichtete [9]. In einer Studie durch Wienhold et al. wurde erstmals in Europa eine telemedizinische Prämedikationsambulanz implementiert und auf technische und medizinische Machbarkeit untersucht [10].

Durch die Coronapandemie wurde die Digitalisierung weltweit erheblich vorangetrieben [11]. Nicht zuletzt durch die branchenübergreifende Schaffung zahlreicher Telearbeitsplätze wurde die Bevölkerung mit dem Thema vertrauter und die IT-Ausstattung deutlich verbessert. Es stellt sich die Frage, welche technische Infrastruktur auf Patientenseite zur Verfügung steht, wie Patienten die telemedizinischen Narkosevorbereitung bewerten und welchen Einfluss die Pandemie auf Infrastruktur sowie die Einstellung des Patienten hat – oder ob sich während der Pandemie nichts verändert hat (Nullhypothese).

## Methoden

### Studiendesign und Statistik

Mithilfe von einem Fragebogen sollen folgende Aspekte erhoben werden, die für eine potenzielle Einführung einer telemedizinischen Narkosevorbereitung relevant erscheinen:

- die persönliche Einstellung potenzieller Nutzer zu einer telemedizinischen Narkosevorbereitung (Akzeptanz und Motivation),
- vorhandene technische Infrastruktur im häuslichen Umfeld des Patienten – u. a. in Bezug auf Videotelefonie-Ausstattung (Technik),
- Einfluss der COVID 19-Pandemie auf Technikverfügbarkeit, -akzeptanz und -motivation.

**Figure 1:**
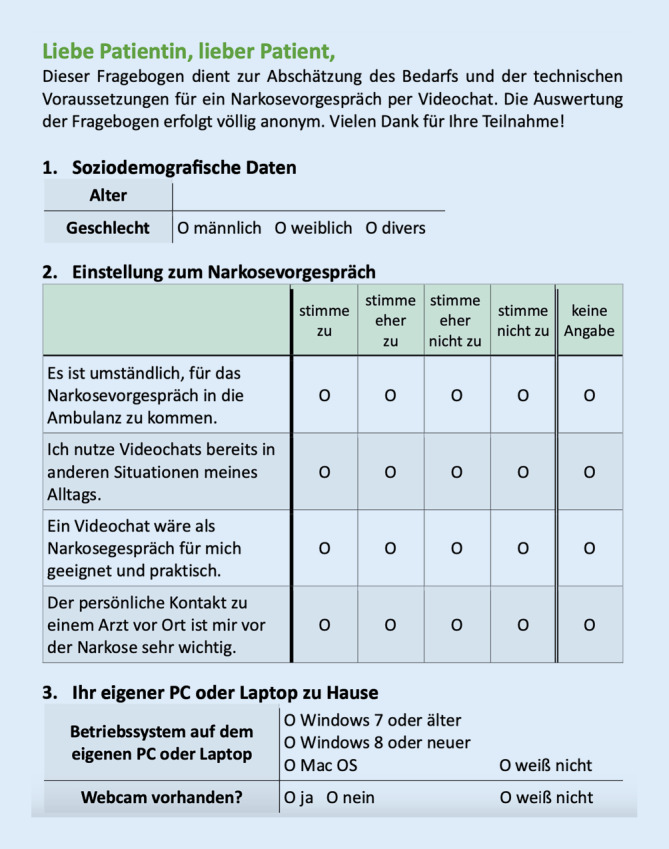

Der Fragebogen umfasste 4 Aussagen zur telemedizinischen Narkosevorbereitung, die von den Ausfüllenden anhand einer 4‑stufigen Likert-Skala (1 = stimme zu; 2 = stimme eher zu; 3 = stimme eher nicht zu; 4 = stimme nicht zu) bewertet wurden (Abb. 1):

- „Es ist umständlich, für das Narkosevorgespräch in die Ambulanz zu kommen.“
- „Ich nutze Videochats bereits in anderen Situationen meines Alltags.“
- „Ein Videochat wäre als Narkosegespräch für mich geeignet und praktisch.“
- „Der persönliche Kontakt zu einem Arzt vor Ort ist mir vor der Narkose sehr wichtig.“

Zusätzlich wurden die Patienten nach einem eigenen PC, dem installierten Betriebssystem sowie dem Vorhandensein einer Webcam befragt.

Zunächst wurde in den Monaten August bis September 2019 eine erste Erhebung in der anästhesiologischen Ambulanz der Uniklinik RWTH Aachen durchgeführt. Um mögliche Auswirkungen der Coronapandemie zu erfassen, wurde von April bis Mai 2020 zu Vergleichszwecken eine erneute Erhebung durchgeführt. Die Teilnahme an beiden Umfragen war freiwillig, stand allen Patienten der Prämedikationsambulanz zur Verfügung und wurde durch die lokale Ethikkommission (EK 151/20) genehmigt. Bei minderjährigen Patienten wurden die Erziehungsberechtigten befragt. Das Patientenkollektiv der Aachener Prämedikationsambulanz ist zu ca. nach 20 % ASA I, 40 % ASA II und 30 % ASA III klassifiziert (Stand 2022). Es wurden sämtliche Eingriffsarten und operativen Fachrichtungen berücksichtigt. Der Fragebogen stand lediglich in Deutsch Sprache zur Verfügung, konnte aber auch – falls für das Aufklärungsgespräch anwesend – mit einem Übersetzer ausgefüllt werden.

Die Datenauswertung erfolgt mit SPSS 24 (IBM Corporation, Armonk, NY, USA), Microsoft Excel (Microsoft Corporation, Redmond, WA, USA) und Graphpad Prism (GraphPad Software, LLC, San Diego, CA, USA). Die vergleichende Analyse wird mit „matched-pairs“ – vor bzw. während der Pandemie – basierend auf dem exakten Alter und Geschlecht durchgeführt. Der Vorzeichentest für abhängige Stichproben wird zur Analyse verwendet und die Effektstärke nach Cohen berechnet. Eine multivariante Regression wird für die unabhängigen Faktoren Alter und Geschlecht durchgeführt. Anschließend werden Subgruppenanalysen nach Alterskohorten (Alter 0 bis 20, 20 bis 40, 40 bis 60, 60 bis 80 und 80 bis 100 Jahre) durchgeführt. Die Rangkorrelation nach Spearman wird errechnet, und der zweiseitige *t*-Test mit Welch-Korrektur wird für die Subgruppenanalyse verwendet.

## Ergebnisse

Im August und September des Jahres 2019 wurden insgesamt 1030 Patienten (inklusive Erziehungsberechtigter bei minderjährigen Patienten) der anästhesiologischen Ambulanz der Uniklinik RWTH Aachen befragt. Um mögliche Auswirkungen der Coronapandemie zu erfassen, wurden im April und Mai 2020 weitere 1050 Patientinnen und Patienten befragt (insgesamt *n* = 2080).

Die Patienten waren dabei zwischen 0 (neugeboren) und 91 Jahren alt. Tab. 1 gibt einen Überblick über die demografischen Daten.

**Table 1:** 

	Alter (in Jahren)			Geschlecht			
	Min	Mittel	Max	Männlich	Weiblich	Divers	Keine Angabe
Vor der Pandemie (*n* = 1030)	0	46,1	85	44,8 % (461)	53,3 % (549)	0 % (0)	1,9 % (20)
Während der Pandemie (*n* = 1050)	1,5	44,4	91	38,8 % (407)	55 % (577)	0,2 % (2)	6,1 % (64)

**Figure 2:**
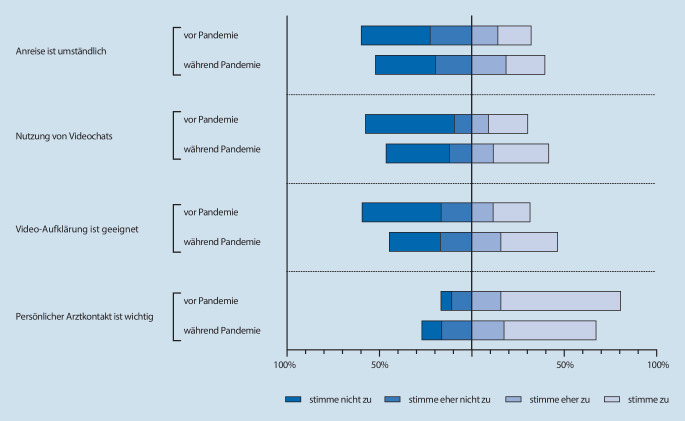

Vor der COVID-19-Pandemie bewerteten 32,3 % der befragten Patienten die persönliche Vorstellung in der anästhesiologischen Ambulanz als (eher) umständlich (*18,0* *% stimme zu, 14,3* *% stimme eher zu*). Während der Pandemie stieg der Anteil an Patienten auf 39,7 % (*21,0* *% stimme zu, 18,7* *% stimme eher zu*) (Abb. 2).

Vor der Pandemie nutzten 30 % der Befragten regelmäßig die Videotelefonie in anderen Alltagssituationen (*21,2* *% stimme zu, 9,2* *% stimme eher zu*)*.* Dieser Anteil stieg während der Pandemie auf 41,8 % (*30,0* *% stimme zu, 11,8* *% stimme eher zu*).

Von den vor der Pandemie Befragten bewerteten 31,7 % die Videotelefonie als geeignet und praktisch für die Vorbereitung auf die Anästhesie (*20,0* *% stimme zu, 11,7* *% stimme eher zu*). Während der Pandemie stieg dieser Anteil auf 46,6 % (*30,7* *% stimme zu, 15,9* *% stimme eher zu*).

Vor der COVID-19-Pandemie hielt die Mehrheit der Teilnehmer den persönlichen Kontakt mit einem Arzt vor der Anästhesie für wichtig (*64,8* *% stimme zu, 15,9* *% stimme eher zu*).

Während der Pandemie war der persönliche Kontakt mit einem Arzt vor der Narkose für die Mehrheit der Patienten (*49,8* *% stimme zu, 17,6* *% stimme eher zu*) nach wie vor von Bedeutung, allerdings weniger wichtig als noch zuvor (80,7 % vs. 67,4 %).

Um die Ergebnisse vor und während der COVID-19-Pandemie zu vergleichen, wurde eine „matched-pairs“-Analyse durchgeführt. Es konnten basierend auf den beiden Kriterien Alter und Geschlecht 630 Paare gebildet werden. Die Analyse der *matched pairs* mit dem Wilcoxon-Rangsummentest zeigt, dass sich die beiden Gruppen, Teilnehmer vor und während der COVID-19-Pandemie, in ihren Antworten in allen 4 Fragen signifikant unterscheiden.

Auf die Frage nach der Umständlichkeit der persönlichen Vorstellung in der anästhesiologischen Ambulanz, empfanden die Patienten vor der COVID-19-Pandemie dies signifikant beschwerlicher als während der Pandemie (z = −2,542, *p* < 0,011). Die Effektstärke nach Cohen ist schwach (r = 0,11).

Videotelefonie wurde im Alltag während der COVID-19-Pandemie signifikant häufiger genutzt als vor der Pandemie (z = −5,335, *p* < 0,001). Auch dieser Effekt ist als schwach (r = 0,23) zu bewerten.

Die Teilnehmer vor der Pandemie empfanden ein Narkosevorgespräch in Form einer Videokonferenz signifikant häufiger als ungeeignet und unpraktisch als die Teilnehmer während der Pandemie (z = −6,678, *p* < 0,001). Dies ist nach Cohen ein mittlerer Effekt (r = 0,28).

Weiterhin wird der persönliche Kontakt mit einem Arzt vor der Narkose während der Pandemie als weniger wichtig angesehen als vor der Pandemie (z = −4,994, *p* < 0,001). Auch dieser Effekt ist als schwach zu bewerten (r = 0,21).

Ob der Patient Videokonferenzen im Alltag nutzte, stand bereits vor der Pandemie in direktem Zusammenhang mit dem Alter (ρ = 0,288, *p* < 0,001). Gleiches galt für die Aussagen, ob der Patient Videokonferenzen als Narkosevorgespräch praktisch fand (ρ = 0,236, *p* < 0,001), und ob der Patient es wichtig fand, vor der Narkose persönlichen Kontakt zum Arzt zu haben (ρ = −0,225, *p* < 0,001).

Während der Pandemie korrelierte die Nutzung von Videokonferenzen (ρ = 0,249, *p* < 0,001) weiterhin mit dem Alter, ebenso wie die Tatsache, ob Patienten Videokonferenzen praktisch und geeignet empfanden (ρ = 0,223, *p* < 0,001). Die Meinung zur Eignung einer Videokonferenz für ein Anästhesievorgespräch (ρ = 0,138, *p* < 0,001) sowie die Wichtigkeit des persönlichen Kontakts zum Arzt (ρ = −0,220, *p* < 0,001) korrelierten ebenfalls zum Alter.

Zwischen der Aussage „Es ist umständlich, für das Narkosevorgespräch in die Ambulanz zu kommen“ und dem Alter bestand kein statistischer Zusammenhang.

**Figure 3:**
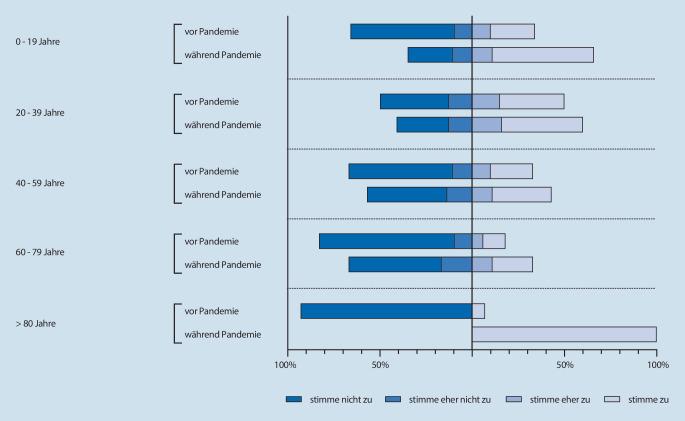

**Figure 4:**
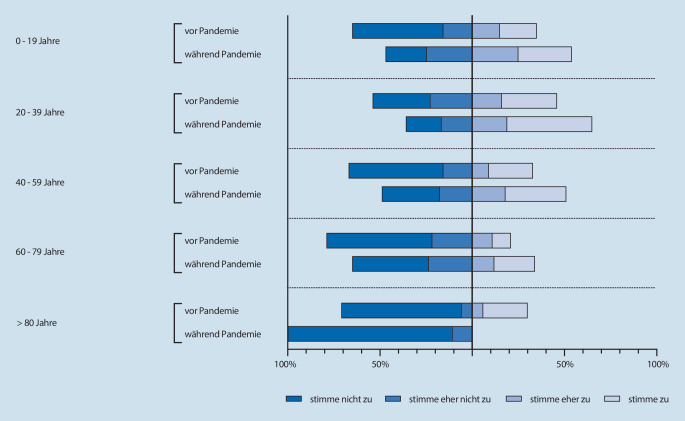

Aufgeteilt nach Alterskohorten nahmen – sofern das Alter angegeben wurde – an der Befragung 129 Personen im Alter von 0 bis 19 Jahren (61 vor und 68 während der Pandemie), 536 Personen im Alter von 20 bis 39 Jahren (245 vor und 291 während der Pandemie), 608 Personen im Alter von 40 bis 59 Jahren (299 vor und 309 während der Pandemie), 371 Personen im Alter von 60 bis 79 (199 vor und 172 während der Pandemie) sowie 30 Personen im Alter von 80 Jahren oder älter (20 vor und 10 während der Pandemie) teil. Die nachfolgenden Abbildungen zeigen die Bewertung der Aussagen „Ich nutze Videochats bereits in anderen Situationen meines Alltags“ (Abb. 3) und „Ein Videochat wäre als Narkosegespräch für mich geeignet und praktisch“ (Abb. 4) nach diesen Alterskohorten.

Tab. 2 zeigt die Ergebnisse der multivarianten Regression der Variablen Altern und Geschlecht auf die 4 Aussagen im Fragebogen.

**Table 2:** 

		Anreise ist umständlich		Nutzung von Videochats		Videoaufklärung ist geeignet		Persönlicher Arztkontakt ist wichtig	
		β	*p*	β	*p*	β	*p*	β	*p*
Alter	Vor der Pandemie	0,001	0,889	0,019	*<* *0,001*	0,016	*<* *0,001*	−0,010	*<* *0,001*
	Während der Pandemie	0,008	*<* *0,001*	0,020	*<* *0,001*	0,016	*<* *0,001*	−0,012	*<* *0,001*
Geschlecht	Vor der Pandemie	0,152	0,069	0,168	0,066	0,343	*<* *0,001*	−0,122	0,055
	Während der Pandemie	−0,034	0,686	0,221	*0,018*	0,155	0,074	0,028	0,717

Die Subgruppenanalyse der Alterskohorten ergab, dass die Nutzung von Videotelefonie im Alltag sowohl vor als auch während der Pandemie mit dem Alter der Patienten korrelierte. Die Nutzung nimmt während der Pandemie in den Alterskohorten der 20- bis 39-Jährigen sowie der 60- bis 80-Jährigen zu (vor vs. während Pandemie: < 20 Jahre: *p* = 0,068; 20 bis 39 Jahre: *p* = 0,0186; 40 bis 59 Jahre: 0,347; 60 bis 79 Jahre: *p* = 0,0004; > 80 Jahre: *p* = 0,345).

Während der Pandemie wurde dem persönlichen Kontakt in den Alterskohorten der 20- bis 79-Jährigen weniger Bedeutung zugemessen wurde also noch vor der Pandemie (vor vs. während Pandemie: 20 bis 39 Jahre: *p* < 0,0001; 40 bis 59 Jahre: *p* < 0,0001; 60 bis 79 Jahre: *p* < 0,001).

Tab. 3 gibt einen Überblick über die vorhandene IT-Infrastruktur auf Patientenseite.

**Table 3:** 

	PC oder Notebook				Webcam verfügbar		
	Windows		Mac	Keine Angabe/kein PC	Ja	Nein	Keine Angabe/keine Webcam
	Version 7 oder älter	Version 8 oder neuer					
Vor der Pandemie (*n* = 1030)	17,7 % (182)	47,9 % (493)	8,8 % (91)	25,6 % (264)	50,4 % (519)	37,6 % (387)	12 % (124)
Nach der Pandemie (*n* = 1050)	11,6 % (122)	57,5 % (604)	8,9 % (93)	22 % (231)	58,2 % (611)	30,6 % (321)	11,2 % (118)
Total (*n* = 2080)	14,6 % (304)	52,7 % (1097)	8,8 % (184)	23,9 % (495)	54,3 % (1130)	34 % (708)	11,7 % (242)

## Diskussion

Durch eine vergleichende Querschnittanalyse vor und während der Pandemie wurde die Einstellungen der Patienten einer telemedizinischen Narkoseaufklärung gegenüber sowie die technischen Voraussetzungen auf Patientenseite evaluiert. Die Auswertung der Befragung von 2080 Patienten zeigt einerseits eine gesteigerte Bereitschaft der Patienten zu einer telemedizinischen Narkosevorbereitung und andererseits durch die COVID-19-Pandemie verbesserte technische Voraussetzungen auf Patientenseite für die Implementation einer telemedizinischen Prämedikationsambulanz.

Die Akzeptanz eines Narkosevorgesprächs in Form einer Videokonferenz stieg durch die COVID-19-Pandemie deutlich an. Während der Pandemie bewerteten mehr Patienten die persönliche Vorstellung in der anästhesiologischen Ambulanz als umständlich (32,3 % vs. 39,7 %), während der persönliche Kontakt zu einem Arzt vor Ort an Bedeutung verlor (80,7 % vs. 67,4 %). Auch wenn die Akzeptanz der telemedizinischen Patientenaufklärung über alle Altersgruppen hinweg zunahm (31,7 % vs. 46,6 %), besteht ein signifikanter Zusammenhang zwischen dem Alter der Patienten und der Akzeptanz einer präoperativen Beratung und Aufklärung per Videotelefonie. Ältere Patienten neigen erwartungsgemäß dazu, Videochats im Alltag seltener zu nutzen und halten eher am persönlichen Kontakt mit dem Arzt fest. Da die Narkosevorbereitung v. a. auch eine vertrauensbildende Maßnahme im Hinblick auf die Narkose ist, gilt es diese Alterskohortendifferenzen zu berücksichtigen, und eine telemedizinische Narkosevorbereitung v. a. bei älteren Patienten als freiwilliges Angebot neben dem Vor-Ort Gespräch, nicht aber als einzige Option zu realisieren.

Es mag verwundern, dass nicht mehr Patienten den Besuch in der Prämedikationsambulanz als umständlich bewerteten. Es kommt hier häufig zu langen Wartezeiten [2], und gerade jüngere Patienten müssen dies mit ihrem Arbeitsalltag koordinieren, während die Anreise für ältere Patienten mitunter beschwerlich ist. Zudem scheinen besonders im Rahmen der COVID-19-Pandemie eine Reduktion persönlicher Kontakte sowie die Vermeidung von Warteräumen und der Nutzung des öffentlichen Personennahverkehrs (ÖPNV) sinnvoll. Die telemedizinische Narkosevorbereitung kann hier zur Unterbrechung von Infektionsketten beitragen.

Während der Pandemie konnten zahlreiche Arbeitnehmer im Homeoffice ihrer Arbeit nachgehen; die Quote hat 25 % erreicht [12]. In diesem Zusammenhang wurden ihre Arbeitsplätze entsprechend ausgestattet. Dies betraf auch die befragten Patienten, die über eine verbesserte technische Infrastruktur verfügten und diese auch vermehrt im Alltag nutzten. Somit ebnet die Pandemie den Weg für die Einführung einer telemedizinischen Narkosevorbereitung.

Durch eine zunehmende Vertrautheit mit modernen Medien sowie mit der Methode der Videotelefonie war es für mehr Patienten denkbar, diese für eine Narkosevorbereitung zu nutzen – ohne dass die Möglichkeiten einer solchen Verfahrensweise bekannt oder den Patienten erklärt wurde. Dabei kann eine telemedizinische Narkosevorbereitung nicht nur das reine Gespräch umfassen, sondern impliziert auch das digitale Ausfüllen des Anamnesebogens [10] mit einem abschließenden Plausibilitätscheck und perspektivisch verschiedenen Möglichkeiten der Integration einer künstlichen Intelligenz. Auch die Erläuterung von Narkoseverfahren, z. B. über Videos, sowie eine teilautomatisierte Dokumentation können in Zukunft die personellen Ressourcen in anästhesiologischen Kliniken schonen und die Qualität der Narkosevorbereitung steigern [5].

Die Mehrheit der Patienten betonte in der Befragung die Bedeutung eines persönlichen Kontaktes vor der Narkose (80,7 % vs. 67,4 %). Diese Bedeutung sank während der Pandemie, vermutlich auch, weil in anderen medizinischen Fachbereichen telemedizinische Konzepte etabliert wurden. Dieser Punkt ist entscheidend für die Akzeptanz einer telemedizinischen Narkosevorbereitung auf Patientenseite. Auch wenn über einen Videochat in offenen Gesprächen Risiken erläutert und ein empathisches Einwirken auf die Ängste der Patienten möglich ist, muss mit einer initialen Ablehnung gerechnet werden. Umso wichtiger sind daher eine intuitive Bedienung für den Patienten, eine umfangreiche Information über den Ablauf sowie die Option, sich bei Unsicherheit für ein konventionelles Gespräch in der anästhesiologischen Ambulanz („face to face“) umentscheiden zu können.

In der Alterskohorte der > 80-Jährigen fällt eine abnehmende Zustimmung zum Videochat als Narkosegespräch auf. Hier zeigt sich, dass eine telemedizinische Risikoaufklärung nicht für alle Patientengruppen geeignet sein wird, sondern lediglich eine Ergänzung zum Angebot des persönlichen Gespräches in den Ambulanzen sein kann. Individuell erhöhte Risiken aufgrund chronischer Erkrankungen wollen von Betroffenen eher persönlich besprochen werden. Darüber hinaus haben gerade diese Altersgruppen in der Pandemie auch unter Einsamkeit gelitten und den persönlichen Kontakt vermisst. Technische Hürden müssen berücksichtigt werden. Zu diesen Erkenntnissen kommt auch ein Leitthemenbeitrag, der die Anforderungen, den Stand der Technik sowie medikolegale und rechtliche Aspekte betrachtet hat [13].

Bisherige Konzepte sahen häufig lediglich eine telemedizinische präoperative Evaluation im klinischen Kontext, nicht aber aus dem direkten häuslichen Umfeld des Patienten vor [14]. So werden Pflegekräfte in Seniorenheimen mit einem Rollwagen eingesetzt, auf dem beispielsweise eine Kamera für die Racheninspektion zur Mallampati-Klassifikation installiert ist [15]. Die Pflegekraft kann dann auch andere Untersuchungen vor Ort durchführen, während der Anästhesist über den Videochat die mündliche Aufklärung übernimmt. Würde man eine telemedizinische Narkosevorbereitung aus dem häuslichen Umfeld der Patienten konzipieren, so entfielen damit alle Möglichkeiten der körperlichen Untersuchung. Zwar könnte man bei guter Beleuchtung und hochauflösender Kamera eine Racheninspektion vornehmen, allerdings müsste man sich bezüglich möglicher Belastungszeichen wie peripherer Beinödeme auf die Einschätzung des Patienten verlassen. Abschätzungen eines kardiopulmonalen Risikos müssten verstärkt in die Anamneseerhebung integrieren werden. Eine Auskultation kann ohne ein digitales Stethoskop beim Patienten aktuell nicht telemedizinisch sinnvoll umgesetzt werden, derartige Befunde könnten bei einer körperlichen Untersuchung jedoch auch bei Aufnahme des Patienten präoperativ erhoben werden. Wie häufig diese unmittelbar präoperative Untersuchung durch Erhebung narkoserelevanter Befunde zu einer Verzögerung der Operation führt, muss Gegenstand weiterer Forschung sein.

Die gewonnenen Informationen bezüglich der vorhandenen Infrastruktur geben einen Aufschluss über die möglichen technischen Spezifikationen, die einer telemedizinischen Software zugrunde liegen sollten. Hier zeigt sich, dass zwar die Mehrheit der Patienten über moderne Endgeräte zu verfügen scheint, jedoch nur knapp die Hälfte eine Webcam besitzt. Der hohe Prozentsatz an fehlenden Antworten zeigt, dass eine Kenntnis über das vorhandene Betriebssystem nicht zwingend vorausgesetzt werden kann. Unklar bliebt, ob einige über eine Webcam verfügen (z. B. integriert in einen Laptop), ohne es zu wissen. Alternativ könnte eine App auch so programmiert werden, dass sie auf mobilen Endgeräten mit integrierter Kamera wie Smartphones und Tablet-PC genutzt werden kann. Um eine breite Nutzbarkeit auf verschiedenen Betriebssystemen zu ermöglichen, sollte die Entwicklung einer Web-Applikation in Betracht gezogen werden.

Einige Limitationen sind zu beachten. Fremdsprachige Befragte konnten nur mit einem Übersetzer teilnehmen, obwohl eine digitale Unterstützung zur Narkosevorbereitung auch in mehreren Sprachen verfügbar gemacht werden könnte. Diese Umfrage repräsentiert nur einen kurzen Zeitraum während der Pandemie. Eine weitere Umfrage mit einer noch größeren Stichprobe wäre sinnvoll, um die weitere Entwicklung während und nach der Pandemie abzubilden. Diese Befragung kann nur eine erste Einschätzung zur Akzeptanz und zur technischen Nutzung durch die Patienten geben. Auch wurden in dieser rein anonymen Patientenbefragung weder ASA-Klassifikationen noch Diagnosen berücksichtigt, weshalb die Kriterien der *matched pairs* eingeschränkt waren. Hier müssen Implementierungsstudien zeigen, wie sich unterschiedliche Patientenrisiken auf die Akzeptanz auswirken.

Eine erste Studie zeigte nach der Konzeption und Entwicklung einer Softwarelösung zur telemedizinischen Narkosevorbereitung bereits die Machbarkeit in Europa [10], eine randomisierte kontrollierte Studie muss jedoch noch folgen. Es ist zu erwarten, dass nach einer verständlichen Information der Patienten ein neues telemedizinisches Konzept und dessen Nutzung von deutlich mehr Patienten anerkannt werden.

## Fazit

- Bereits jetzt hält fast die Hälfte der Patienten eine telemedizinische Durchführung der Narkosevorbereitung für geeignet und praktisch.
- Im Hinblick auf Digitalisierung und technische Infrastruktur ebnet die COVID-19-Pandemie den Weg für eine telemedizinische Narkosevorbereitung.
- Durch die verstärkte Nutzung der Videotelefonie im Alltag während der Pandemie hat sich die Einschätzung der Praktikabilität von Videochats als Vorbereitung auf die Anästhesie verbessert. Gerade jüngeren Patienten kann ein solches Konzept lange Wartezeiten und unnötige Anfahrten in die Prämedikationsambulanz ersparen.
- Die Auswirkungen einer körperlichen Untersuchung am Operationstag auf den perioperativen Ablauf sowie die Rechtskonformität sollten erfasst und evaluiert werden.
- Mögliche Vorteile einer telemedizinischen Narkosevorbereitung umfassen neben den Vorteilen auf Patientenseite einen zielgerichteten Ressourceneinsatz durch kürzere Gesprächszeiten und eine Optimierung der Dokumentation in den Kliniken.
